# The Reliability of Child-Friendly Race-Attitude Implicit Association Tests

**DOI:** 10.3389/fpsyg.2016.01576

**Published:** 2016-10-24

**Authors:** Amanda Williams, Jennifer R. Steele

**Affiliations:** ^1^Graduate School of Education, University of Bristol, BristolUK; ^2^Department of Psychology, York University, Toronto, ONCanada

**Keywords:** psychometrics, test reliability, implicit attitudes, children, IAT

## Abstract

Implicit attitudes are evaluations that are made automatically, unconsciously, unintentionally, or without conscious and deliberative processing (Nosek et al., 2007; Gawronski and De Houwer, 2014). For the last two decades implicit measures have been developed and used to assess people’s attitudes and social cognition, with the most widely used measure being the Implicit Association Test (IAT; Greenwald et al., 2003). This measure has been used extensively to assess racial biases and a number of studies have examined the reliability of the IAT when administered to adults (Cunningham et al., 2001; Gawronski, 2002; Greenwald et al., 2003; Nosek et al., 2005; Nosek and Smyth, 2007; Bar-Anan and Nosek, 2014). In recent years, the IAT has also been modified for use with children. Despite the potential of this measure to provide insight into the early emergence of implicit racial attitudes, little is known about the psychometric properties of these modified child-friendly IATs (Child-IATs). In the current research we examined the internal consistency of race-attitude Child-IATs when either reduced (Study 1) or traditional-length (Study 2) versions were administered to children (Studies 1 and 2) and adults (Study 2). We also examined the test–retest reliability of this measure with both child and adult participants (Study 2). We found that these measures demonstrate an internal consistency comparable to what has been seen in previous research with adults. In addition, the internal consistency of traditional-length Child-IATs completed in succession depended on the order in which they were completed; the first Child-IAT demonstrated higher internal consistency than the second for both children and adults (Study 2). Finally, we provide the first evidence that the test–retest reliability of the Child-IAT is comparable to what has been found previously with adults (Study 2). The implications of these findings for future research examining children’s implicit social cognition are discussed.

## Introduction

Despite historical decreases in the explicit endorsement of overt racism (Dovidio et al., 2009), majority group members continue to express subtle forms of racial bias. One potential explanation for this discrepancy is the limitations of explicit measures; responses can be biased by self-presentation concerns and are limited by introspection (see Teige-Mocigemba et al., 2010; Nosek et al., 2011, for reviews). To address these concerns, researchers have developed measures to assess *implicit* racial attitudes, that is the uncontrollable, associative, and automatically activated evaluations that are believed to underlie our thoughts and behaviors (Lane et al., 2007; Payne and Gawronski, 2010; Teige-Mocigemba et al., 2010; Nosek et al., 2011). Supporting their role in the persistence of racial inequalities, implicit attitudes have been found to predict behavior during intergroup interactions above and beyond explicit attitudes (see Greenwald et al., 2009; Cameron et al., 2012, for meta-analyses).

Because of their importance for understanding intergroup relations, a great deal of social psychological theory and research in the past two decades has focused on implicit racial attitudes. One of the earliest measures developed to assess these attitudes is the Implicit Association Test (IAT; Greenwald et al., 1998). The IAT is a computer-based reaction-time measure which compares the speed with which participants pair target-concepts (e.g., Black-American vs. White-American) with attributes (e.g., pleasant vs. unpleasant stimuli) across two critical blocks. On this measure, people who hold an implicit pro-White (relative to Black) bias are faster at pairing White targets with pleasant stimuli and Black targets with unpleasant stimuli, relative to the reverse pairing.

Since its creation, the IAT has been used to examine a wide variety of implicit social cognitions among adults, with racial attitudes being among the most frequently assessed (Teige-Mocigemba et al., 2010; Nosek et al., 2011). An important factor contributing to the popularity of the IAT for social psychologists is its relatively strong psychometric properties, particularly in comparison to other implicit measures (e.g., Cunningham et al., 2001; Bar-Anan and Nosek, 2014). Recently, researchers have started to create child-friendly versions of the IAT (Child-IAT) to assess children’s implicit racial attitudes; however, there is limited information regarding the psychometric properties of this modified measure. In the present study we seek to extend this literature by examining the reliability of the Child-IAT.

### Children’s Racial Attitudes

Researchers are increasingly interested in the emergence of implicit social cognition in childhood. As such, the race-attitude IAT has been modified for use with children (see Olson and Dunham, 2010; McKeague et al., 2015, for reviews) and has been used to assess children’s implicit racial biases in North America (e.g., Baron and Banaji, 2006; Newheiser and Olson, 2012) and beyond (e.g., Rutland et al., 2005; Dunham et al., 2006; Newheiser et al., 2014). Younger White children typically show racial preferences favoring their own racial ingroup on both implicit and explicit measures. However, by at least 8 years of age, implicit and explicit racial attitudes have been found to diverge. Despite a marked decrease in explicit racial bias that typically occurs by the age of 8-years (see Raabe and Beelmann, 2011, for a review), implicit racial bias as measured by the IAT remains stable across childhood and into adulthood (Rutland et al., 2005; Baron and Banaji, 2006). With consistency, children demonstrate relative implicit preference for ingroups and dominant outgroups relative to less dominant outgroups (see Olson and Dunham, 2010, for a review), often at a magnitude that is comparable to the implicit racial preferences of adults.

In addition, as with adults, the few studies that have examined children’s behavior suggest that responses on Child-IATs may be meaningfully related to intergroup behavior. White 8- to 11-year olds who displayed more positive racial outgroup attitudes on the IAT tended to report more cross-race friendships (Turner et al., 2007) and primarily White 4- to 6-year-olds who demonstrated stronger implicit bias favoring their minimal ingroup relative to a minimal outgroup allocated more resources (coins) to minimal ingroup members (Dunham et al., 2011). Although more research is needed, these findings suggest that racial biases as measured by the Child-IAT may meaningfully predict children’s behavior in their interactions with racially diverse peers.

Although results with adults, and to some extent children, support the predictive validity of the IAT, little is known about the psychometric properties of the Child-IAT when administered to children or adults. It is important to examine the reliability of the race-attitude Child-IAT if we wish to further predict children’s intergroup behavior and/or assess the effectiveness of prejudice-reduction interventions. For example, if an intervention alters implicit racial attitudes, then we have a greater chance of identifying this as an effective approach when using psychometrically sound, reliable measures as assessment tools. Although the adult version of the IAT has strong psychometric properties, “it cannot be assumed that there would be similar rates of reliability and validity in these measures with children” (Olson and Dunham, 2010, p. 251). Given the increasing popularity of Child-IATs, as an initial investigation into its psychometric properties, we first review the existing information on the reliability of Child-IATs. Second, through two studies we examine the reliability of race-attitude Child-IATs when completed by both children and adults, with a specific focus on (1) internal consistency and (2) test–retest reliability.

### Internal Consistency of the IAT

Reliability provides an indication of the dependability of a measure by identifying the proportion of total observed variance that reflects consistent (or true) as compared to random error variance (Cronbach, 1951). Internal consistency, which refers to the degree to which responses on a measure are consistent or related to one another, is one method used to assess reliability. Measures with low internal consistency, or a high degree of measurement error, may not provide useful information as scores will be unpredictable and not extend to meaningful interpretations of test performance (Cronbach, 1951). Further, measures with low internal consistency will have attenuated effects, such as limited correlations with other measures (Nunnally, 1978; Cunningham et al., 2001) and reduced effect sizes (Baugh, 2002), which can limit researchers’ ability to appropriately interpret their results. By using psychometrically sound measurement tools, researchers will be in a better position to comment on the potential disassociation between implicit and explicit attitudes, and when this might occur in development (e.g., Rutland et al., 2005).

Researchers reporting the internal consistency of IATs typically present split-half correlations (Spearman–Brown corrected) between *D*-scores calculated separately for “practice” and “test” trials from the critical blocks presenting the combined attribute and target discrimination task (Greenwald et al., 2003). However, this approach may be limited as different methods of splitting the data (e.g., practice vs. test trials, even vs. odd trials, etc.) can produce different internal consistency coefficients (Cronbach, 1951; Nunnally, 1978). By contrast, coefficient alpha is, by mathematical definition, the average of all the possible split-half coefficients (Cronbach, 1951). In line with recommendations that coefficient alpha be used to assess the internal consistency of new measures (Nunnally, 1978), researchers who have specifically investigated the psychometric properties of the IAT with adults have often used coefficient alpha to estimate internal consistency (e.g., Bosson et al., 2000; Cunningham et al., 2001; Gawronski, 2002; Gschwendner et al., 2008). Because including more items in the analyses can result in higher coefficient alpha, these researchers have used trial-based difference scores or scores based on test quarters as items in their analyses. For these reasons, we also use coefficient alpha based on test quarters to estimate the internal consistency of children’s responses.

Research examining the internal consistency of race-attitude IATs in adult samples have reported coefficient alphas that range from 0.55 to 0.88 (Cunningham et al., 2001; Gawronski, 2002; Nosek and Smyth, 2007; Bar-Anan and Nosek, 2014) and split-half reliabilities from 0.43 to 0.67 (Greenwald et al., 2003; Nosek et al., 2005). Although not as high as what is typically found for self-report measures, the internal consistency of the IAT falls within the acceptable range for reaction time measures which, by their very nature, show greater fluctuation across trials (Cunningham et al., 2001; Nosek et al., 2011). The reason for increased response variability on implicit measures could stem from a variety of sources, including having a larger response range (i.e., ranging 0 to 10,000 ms on an implicit measure as compared to 1 to 7 on an explicit measure), momentary inattention to the task (i.e., blinking, sneezing), and/or participants adopting different response strategies within the task (Buchner and Wippich, 2000; De Houwer, 2003; Fiedler and Bluemke, 2005; Lane et al., 2007). Such sources of error variance do not similarly influence responses on explicit measures. Although the IAT is not without its limitations (a point returned to in the General Discussion), it is worth noting that this measure is generally found to have stronger internal consistency as compared to other measures of implicit social cognition (e.g., Bosson et al., 2000; Cunningham et al., 2001; Degner and Wentura, 2010; Bar-Anan and Nosek, 2014), a fact that has contributed to its continued use and popularity.

To date, only a small proportion of published studies report any internal consistency information when race-attitude Child-IATs are completed by children. In two of the papers that reported internal consistency, coefficient alphas ranged from 0.73 to 0.81 (Turner et al., 2007; Zezelj et al., 2015). In other studies, split-half correlations ranged from 0.55 to 0.73 (Degner and Wentura, 2010; Haye et al., 2010; Dunham et al., 2014; see Supplementary Materials for a comprehensive review). These preliminary findings suggest that when administered to children, the internal consistency of race-attitude Child-IATs is comparable to what is found when adults complete a traditional version of this task. However, given the increasing use of this measure with children, a more systematic investigation into the internal consistency and stability of race-attitude Child-IATs is needed (Olson and Dunham, 2010; McKeague et al., 2015).

The goal of the present research was to systematically examine the internal consistency and test–retest reliability of race-attitude Child-IATs completed by 5- to 11-year-olds and by adults. To replicate the conditions under which these tasks are frequently administered, we examined the reliability of the race-attitude Child-IAT when (a) the measure was reduced in length (Study 1) and (b) two Child-IATs were completed in succession (Study 2).

#### Reduced-Length Child-IAT

To accommodate limited attention spans and testing time, a common modification is to reduce the number of critical trials within the task (reduced-length IATs; e.g., Rutland et al., 2005; Dunham et al., 2014). The only study with an adult sample to report the internal consistency of a reduced-length race-attitude IAT (e.g., Brief-IATs; Sriram and Greenwald, 2009; Nosek et al., 2014) found the internal consistency to be 0.81 (Bar-Anan and Nosek, 2014). However, it is unclear whether reducing the length would affect the internal consistency of Child-IATs completed by children. It seems possible that presenting children with a reduced measure may lead to *better* attention throughout the duration of the task, which would limit error variance and improve internal consistency. On the other hand, children may show increased variability in responding in general, and therefore more trials may be required in order to capture their true score variance (Cronbach, 1951; Nunnally, 1978). To examine these possibilities, in the present research we administered both reduced- (Study 1) and traditional- (Study 2) length IATs.

#### Internal Consistency When IATs Are Completed in Succession

In research with children, limited access to participants can lead to the administration of more than one Child-IAT within a single session (e.g., Baron and Banaji, 2006; Andrews et al., 2010; Cvencek et al., 2011; Cvencek et al., 2014). Yet it is unclear whether responses on subsequently completed Child-IATs are as internally consistent as the first. It is possible that additional sources of error (e.g., fatigue, learning effects) unrelated to true score variance are introduced when completing Child-IATs in succession, thus decreasing the internal consistency on repeated administrations. This is an important consideration for researchers aiming to assess the effectiveness of prejudice-reduction interventions and/or examine relationships with Child-IATs as measures with low internal consistency can have attenuated effects (Nunnally, 1978; Buchner and Wippich, 2000; Cunningham et al., 2001). From the few studies that have examined the internal consistency of IATs completed in succession by adults, there is some evidence to suggest that estimates of internal consistency are lower, albeit sometimes only slightly, after the first IAT. For example, the internal consistency of four race-attitude IATs each separated by 2 weeks ranged from α = 0.88 at Time 1, 0.78 at Time 2, 0.75 at Time 3, to 0.68 at Time 4 (Cunningham et al., 2001). A similar pattern was found for different variations of race-attitude IATs administered within 2 weeks of each other (Cunningham et al., 2001; Gschwendner et al., 2008). Of greater relevance to the current research, when slightly different race-attitude IATs were completed in the same testing session, the internal consistency of the first (α = 0.76; White-Latino IAT) was similarly higher than the second (α = 0.60; White-Black IAT; Blair et al., 2010).

The limited research examining this question with children has found a similar pattern. For example, the internal consistency of a smoking/healthy foods Child-IAT was initially 0.54, but was found to be 0.41 1 week later (cf. smoking/sweets Child-IAT; Andrews et al., 2010). And on an aggressiveness Child-IAT separated by intervals of approximately 4 months, the internal consistency dropped slightly between Time 1 (Guttman’s split-half *r* = 0.78) and Times 2 and 3 (*r*s = 0.74; Gollwitzer et al., 2007). In order to examine whether a similar pattern would be found with race-attitude Child-IATs, we examined internal consistencies when the task was completed twice in the same session (Study 2).

### Test–Retest Reliability of the IAT

A second main goal of this research was to examine the test–retest reliability of race-attitude Child-IATs. To the extent that the construct of interest is stable, a measure should yield similar scores across time and these scores should be reliably correlated (e.g., test–retest correlations; Nunnally, 1978; Bosson et al., 2000). Although reported less frequently, researchers examining the test–retest reliability of race-attitude IATs completed by adults have found Pearson *r*s ranging from 0.17 to 0.50 (Cunningham et al., 2001; see also Lane et al., 2007; Gschwendner et al., 2008; Bar-Anan and Nosek, 2014). On other versions of the Child-IAT administered to children, test stability has been comparable to that of the traditional race-attitude IAT completed by adults; test–retest *r*s ranged from 0.20 to 0.70 for smoking attitudes (Andrews et al., 2010) and from 0.14 to 0.39 for aggressiveness (Gollwitzer et al., 2007; Lemmer et al., 2015). In the present research we provide the first examination of the test–retest reliability of the race-attitude Child-IAT.

### The Present Study

The goal of this paper was to examine the psychometric properties of modified race-attitude Child-IATs. To examine whether the length of the measure would impact internal consistency, in Study 1 we administered a reduced-length White–Black race-attitude Child-IAT (Rutland et al., 2005) to children. In Study 2, we examined the internal consistency of a traditional-length (Greenwald et al., 2003) White–Black Child-IAT completed by both children and adults. In addition, in Study 2 we examined (a) whether the internal consistency of a second White–Black Child-IAT completed in the same testing session was comparable to the first, and (b) the test–retest reliability of this measure. In Study 2, we included an adult sample to determine whether the psychometric properties of children’s responses would be comparable to those made by adults. This research is an initial step in addressing the void in the literature regarding the psychometric properties of this increasingly popular race-attitude Child-IAT.

## Study 1

Researchers have created child-friendly IATs by reducing the number of critical trials from 120 to as few as 40 (Dunham et al., 2014), and some initial studies have provided information about the psychometric properties of reduced-length Child-IATs. For example, the internal consistency of reduced-length smoking-attitude Child-IATs had coefficient alphas that ranged from 0.41 to 0.54 (Andrews et al., 2010). The internal consistency of reduced-length race-attitude Child-IATs, which were reported as split-half *r*s, ranged from 0.55 to 0.73 (Degner and Wentura, 2010; Dunham et al., 2014). To contribute to this growing body of knowledge, in Study 1 we examined whether a reduced-length race-attitude Child-IAT would show comparable internal consistency to what has previously been found in adult and child samples. Unlike this previous research, we calculated coefficient alpha as a more accurate estimate of the internal consistency of children’s responding (Cronbach, 1951; Nunnally, 1978).

### Method

#### Participants and Procedure

As part of a larger study, 209 children from a large North American city completed a reduced version of a White–Black Child-IAT (reduced-length Child-IAT). Sixteen children were removed from the analyses, five because of inattention (e.g., would not focus on the task, randomly pushed the buttons), four because of comprehension issues, five because of experimenter or technical errors, and two because their testing session was interrupted. The final sample consisted of 193 children (110 girls, 83 boys) from senior kindergarten to grade 5 (age ranged 5 to 11 years, *M*_age_ = 7.6, *SD* = 1.30). The sample consisted of 28% Black, 28% East/Southeast Asian, 21.8% South Asian, 8.3% West Indian, 6.7% White, 4.1% Multiracial, and 3.1% Hispanic participants.

Children were tested individually during school hours in a quiet location within their school. An experimenter read the instructions to the children and remained present during the entire testing session. If children seemed distracted or did not follow instructions (e.g., not focused on the computer, only used one hand to respond) the experimenter re-directed them and kept them on-task. This study was approved by York University’s and Toronto District School Board’s Ethics Review Boards and conformed to the standards of the Canadian Tri-Council Research Ethics and American Psychological Association ethical guidelines. Parental informed consent and child assent was obtained prior to participation.

#### Measures

##### Reduced Child-Friendly Implicit Association Test

This measure was similar to the task used with adults (Greenwald et al., 2003), but it presented only pictorial stimuli and had a reduced number of trials. Target-concepts were represented by matched photographs of White and Black children and attributes were represented by positive and negative line drawings (happy and sad faces; Rutland et al., 2005).

The reduced-length Child-IAT contained five blocks (Rutland et al., 2005). In Block 1 (16 trials) participants practiced discriminating between target-concept images by sorting pictures of four White and four Black children using two computer keys. In Block 2 (16 trials) participants practiced discriminating between attribute images by sorting four positive and four negative line drawings using the same two computer keys. Block 3 (32 trials) included the first of the critical trials; previously seen target-concept and attribute images were presented sequentially and children were asked to sort them using two computer keys. Some children classified White and positive images using one key and Black and negative images using the other key; other children classified Black and positive targets with one key and White and negative targets with the other (counterbalanced between participants). In Block 4 (16 trials) participants re-classified White and Black images using the reverse key assignments. In Block 5 (32 trials) participants completed the second set of critical trials and sorted the category and attribute stimuli with the retrained key assignments (e.g., if White and positive images shared the same response key in Block 3, Black and positive shared the same response key in Block 5). For the purpose of assigning trials equally to subblocks when calculating internal consistency and creating *D*-scores (see Data Preparation below), in Blocks 3 and 5 the first 12 critical trials were designated at “practice” and the latter 20 critical trials as “test” trials (Greenwald et al., 2003).

The order of critical blocks was counterbalanced between participants (see Supplementary Materials for additional information). In each block, stimuli were presented in random order and both target-concept and attribute images appeared in an equal number of trials. Headers remained on-screen throughout the task to remind participants of the correct response keys. Feedback was not provided for incorrect responses but a correct response was required to move the task forward; correct response latencies were recorded. To reduce the potential effect of gender biases, children were presented with same-sex pictures of White and Black targets.

##### Data preparation

In order to estimate internal consistency, we separated responses into four subblocks of equal length and critical trials were assigned to subblocks based on the order in which they were completed (Gawronski, 2002; Turner et al., 2007; Gschwendner et al., 2008). For example, the first critical trial completed was assigned to subblock 1, the second to subblock 2, the third to subblock 3, the forth to subblock 4, the fifth to subblock 1, and so on. Each subblock consisted of 16 trials; three “practice” and five “test” critical trials pairing White with positive and Black with negative, and three “practice” and five “test” critical trials pairing Black with positive and White with negative.

A *D*-score was calculated for all of the responses and then for each subblock. First, responses greater than 10,000 ms were removed as was one participant with greater than 10% of response latencies falling below 300 ms (Greenwald et al., 2003). The average of the mean reaction time to the White+positive/Black+negative “practice” trials was subtracted from the Black+positive/White+negative “practice” trials and divided by the pooled standard deviations for the “practice” trials. A similar score was created for the “test” trials. These two scores were then averaged to create one *D*-score for each subblock.

##### Internal Consistency

Coefficient alpha was calculated with each of the four *D*-scores as items within the analysis (Gawronski, 2002; Turner et al., 2007; Gschwendner et al., 2008; Bar-Anan and Nosek, 2014).

### Results and Discussion

Using a sample that was more racially diverse than what is typically seen in research on children’s implicit racial attitudes in North America, coefficient alpha for the reduced-length race-attitude Child-IAT was 0.72. This is comparable to the internal consistency coefficients for adults completing traditional-length versions of the IAT (αs ranged 0.55 to 0.88; Cunningham et al., 2001; Gawronski, 2002; Nosek and Smyth, 2007; Bar-Anan and Nosek, 2014) and children completing reduced versions of similar measures (split-half *r*s ranged 0.55 to 0.73; Degner and Wentura, 2010; Dunham et al., 2014), suggesting that a reduced-length race-attitude Child-IAT demonstrates adequate internal consistency when completed by children. Replicating previous research, one-sample *t*-test comparing the overall *D* score to 0 confirmed that children demonstrated implicit racial bias favoring Whites relative to Blacks (*D* = 0.08, *SD* = 0.39), *t*(191) = 3.15, *p* = 0.002, *d* = 0.23. Taken together, these results suggest that a reduced-length race-attitude Child-IAT shows comparable internal consistency as when the traditional-length IAT is administered to adults.

## Study 2

In Study 2 we administered a traditional-length Child-IAT which presented the number of trials typically completed by adults (e.g., Greenwald et al., 2003). If a longer version of the Child-IAT increases fatigue and corresponding error variance, then coefficient alpha should be lower than what was observed in Study 1. We also included a second race-attitude Child-IAT in the same testing session. The purpose of this was twofold. First, we were interested in whether the internal consistency of a second Child-IAT would be lower than the first, as has been the case with adult participants. Second, published studies have not yet examined the test–retest reliability of race-attitude IATs with child samples (McKeague et al., 2015). To the extent tht the Child-IAT captures true score variance, and implicit racial attitudes are stable over time, stronger test–retest correlations should emerge (Bosson et al., 2000). We were therefore interested in providing an initial investigation of the test stability of this measure. Finally, in Study 2b we administered these measures to a large sample of undergraduates to determine whether the internal consistency of the Child-IAT would be comparable when it was completed by adults.

### Method

#### Participants and Procedure

As part of a larger study, 154 children (Study 2a) and 198 adults (Study 2b) from a large North American city completed two Child-IATs within a single testing session. Prior to any analyses, the data for ten children were removed; five because of inattention (e.g., would not focus on the task) and five because technical error prevented the first Child-IAT from being completed. The final sample for Study 2a consisted of 144 children, including 68 younger children in grade 1 (32 girls and 36 boys, aged 6- or 7-years, *M*_age_ = 6.4 years) and 76 older children in grade 4 (36 girls and 40 boys, aged 9- or 10-years, *M*_age_ = 9.4 years), including 60.4% White, 16% Hispanic, 8.3% East/Southeast Asian, 8.3% Multiracial, 5.6% Black, 0.7% Middle Eastern, and 0.7% South Asian participants. The final sample for Study 2b consisted of 198 undergraduates (150 women, 36 men, 12 not specified, aged 17- to 53-years, *M*_age_ = 20.9 years), including 38.4% Black, 29.3% White, 8.6% South Asian, 7.6% West Indian, 6.6% East/Southeast Asian, 5.6% Multiracial, 1.0% South American, and 1.0% Middle Eastern participants (2% did not provide their racial identification). The data of nine children and seven adults were removed from the analyses involving the second Child-IAT either because they were inattentive (four children) or because technical error prevented this measure from being completed (five children, seven adults).

In Study 2a, children were tested individually in a quiet location within their school. Children were read the instructions and completed the measures while the experimenter was present. A different experimenter administered each of the two Child-IATs. In Study 2b, adults independently completed the study in a testing room on campus with written instructions displayed on the computer screen. All other aspects of the procedure and the measures were identical across Studies 2a and 2b. Participants completed the first Child-IAT, followed by approximately 6 minutes of filler tasks and the second Child-IAT. This study was approved by York University’s and the Toronto Catholic District School Board’s Ethics Review Boards and conforms to the standards of the Canadian Tri-Council Research Ethics and the American Psychological Association ethical guidelines. For children, parental permission and verbal assent was obtained prior to participation; for adult participants, written consent was obtained.

#### Materials

##### Child-friendly Implicit Association Test

The traditional-length Child-IAT was identical to the reduced-length Child-IAT, with the following exceptions. First, similar to adult versions, all participants were presented with the same stimuli; target-concepts were represented by matched photographs of White and Black boys. Second, feedback was provided for incorrect responses; a blue “X” remained on-screen until the correct response moved the task forward. Third, the length of the task was increased to be comparable to the IAT typically used with adults.

Unlike the reduced measure administered in Study 1, but similar to the adult version, the Child-IAT separated the critical trials into “practice” and “test” blocks during administration, resulting in seven blocks in total. In Block 1 (20 trials) participants sorted the target-concept images (pictures of four White and four Black boys). In Block 2 (20 trials) participants sorted the attribute images (four positive and four negative line drawings). Blocks 3 (20 “practice” trials) and 4 (40 “test” trials) presented the first set of critical trials. In Block 5 (20 trials) the target-concept images were re-classified using the reverse key assignments. Blocks 6 (20 trials) and 7 (40 trials) presented the second set of critical trials, the target-concept and attribute images were sorted using the retrained key assignments. The order of the critical blocks was counterbalanced between participants and randomized between the two Child-IATs (see Supplementary Materials).

##### Data preparation

First, an overall *D*-score was calculated for each of the Child-IATs using the responses on all of the critical trials. In line with scoring procedures recommended by Greenwald et al. (2003), responses greater than 10,000 ms were removed as was one participant on the second Child-IAT with greater than 10% of their total response latencies falling below 300 ms. The average of the mean reaction time to the White+positive/Black+negative “practice” trials was subtracted from the Black+positive/White+negative “practice” trials and divided by the pooled standard deviations for the “practice” trials. A similar score was created for the “test” trials. These two scores were then averaged to create one overall *D*-score for the first and a separate *D*-score for the second Child-IAT.

Second, to calculate internal consistency, responses on the critical trials were assigned to four subblocks based on the order in which they were completed (Gschwendner et al., 2008). For each participant, each subblock consisted of 30 trials; 5 “practice” and 10 “test” trials pairing White with positive and Black with negative, and 5 “practice” and 10 “test” trials pairing Black with positive and White with negative. *D*-scores were created for each subblock (Greenwald et al., 2003). Due to a programming error, some participants (35 children and 27 adults in IAT1, 28 children and 37 adults in IAT2) completed four extra trials in Block 6. These trials were assigned to the four subblocks based on the order completed and were also used when calculating the *D*-scores.

##### Internal consistency

Coefficient alpha was calculated using the four *D*-scores as items in the analyses. To examine whether both younger (*M*_age_ = 6-years) and older (*M*_age_ = 9-years) children demonstrated comparable internal consistency, we calculated the internal consistency separately for each age group.

##### Test–retest reliability

The *D*-scores for the first and second Child-IAT completed by children and adults were correlated using Pearson’s correlation coefficient (*r*). Because this approach may be limited in its ability to detect systematic error (e.g., learning effects, fatigue), we include Intraclass Correlation Coefficients (ICCs) as an additional indicator of test stability (Bedard et al., 2000; Weir, 2005).

### Results and Discussion

A first goal of Study 2 was to examine whether the internal consistency of a traditional-length Child-IAT would be comparable to that of the reduced-length Child-IAT administered in Study 1. Coefficient alpha for the first completed Child-IAT was 0.76 for younger and 0.85 for older children (**Table 1**). Although comparable, these slightly exceeded the internal consistency of the reduced-length Child-IAT (0.72; Study 1). Further, the internal consistency of children’s responding was comparable to, but slightly higher than, adults completing the same measure, α = 0.75. We suspect that this may be due to differences in administration procedures; children completed the Child-IAT in the presence of an experimenter who re-directed them as necessary, whereas the adults completed the measure independently. Despite these differences, both children and adults demonstrated internal consistencies that were comparable to published studies with adults (αs ranged from 0.55 to 0.88; Cunningham et al., 2001; Gawronski, 2002; Nosek and Smyth, 2007; Bar-Anan and Nosek, 2014). These findings suggest that reducing the length of the Child-IAT does not improve the internal consistency of this child-friendly measure. Instead, internal consistency may be slightly improved by a longer version of the measure (Cronbach, 1951; Nunnally, 1978).

**Table 1:** Estimates of internal consistency (coefficient alpha) by age of participant and order completed (Study 2).

	Age of participant		
	6-Year-Olds	9-Year-Olds	Adults
First Child-IAT	0.76	0.85	0.75
Second Child-IAT	0.67	0.77	0.74

The second goal of Study 2 was to examine the impact of repeated administrations within a single testing session on the internal consistency of the Child-IAT. For the second Child-IAT, α = 0.67 for younger children, α = 0.77 for older children (Study 2a), and α = 0.74 for adults (Study 2b; **Table 1**). For children, coefficient alpha was lower for the second completed Child-IAT. For adults, coefficient alpha was comparable across both administrations. Considering that two traditional-length Child-IATs were completed in succession, children may have fatigued during the second measure, increasing the error variance and thus decreasing internal consistency. Nevertheless, for each age group, the internal consistency of the first and second Child-IAT was still comparable to published studies with adults (0.55 to 0.88; Cunningham et al., 2001; Gawronski, 2002; Nosek and Smyth, 2007; Bar-Anan and Nosek, 2014).

**FIGURE 1:**
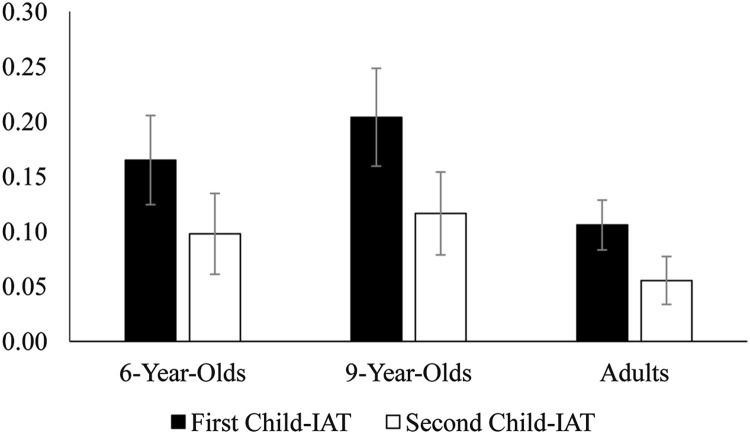
**Mean Implicit Association Test (IAT) *D* scores by age group and order of measure (Study 2).** Error bars represent standard error.

Finally, we examined the test–retest reliability of the two race-attitude Child-IATs completed within a single testing session. One-sample *t*-tests comparing *D*-scores to 0 confirmed that, similar to other research using race-attitude Child-IATs with children, on both Child-IATs, younger children, older children, and adults demonstrated implicit racial bias favoring Whites relative to Blacks, *t*s ≥ 2.53, *p*s ≤ 0.01, *d*s ≥ 0.18, see **Figure 1**. Importantly, paired-samples *t*-tests revealed that the magnitude of bias did not differ between the first and second completed Child-IAT for younger children, *t*(63) = 1.31, *p* = 0.20, *d* = 0.16, older children, *t*(70) = 1.47, *p* = 0.15, *d* = 0.17, or adults, *t*(189) = 1.62, *p* = 0.11, *d* = 0.12. This provides some initial evidence of reliability, as the measure yielded similar scores at both times.

In addition, for both children, *r* = 0.24, *n* = 135, *p* = 0.005 [ICC(2,1) = 0.39], and adults, *r* = 0.33, *n* = 198, *p* < 0.001 [ICC(2,1) = 0.50], significant test–retest correlations and moderate Intraclass Correlation Coefficients emerged, although for children this relationship was slightly lower than for adults. These correlations fall within the lower range of test–retest reliabilities typically seen for adults completing race-attitude IATs (*r*s from 0.17 to 0.50; Cunningham et al., 2001; see Lane et al., 2007, for a review), and are comparable to published studies examining children’s smoking attitudes (0.20 to 0.70; Andrews et al., 2010) and aggressiveness (0.14 to 0.39; Gollwitzer et al., 2007; Lemmer et al., 2015).

## General Discussion

The primary goal of this paper was to determine if the reliability of the race-attitude Child-IAT completed by children is comparable to previously established reliabilities from traditional IATs completed by adults. Based on these two studies, the answer is a resounding “Yes!” Coefficient alphas for our child participants fell within the range found in published studies with adults completing race-attitude IATs (Cunningham et al., 2001; Gawronski, 2002; Nosek and Smyth, 2007; Bar-Anan and Nosek, 2014). In addition, the test–retest reliability was comparable to published studies with adults (Cunningham et al., 2001) and studies examining smoking attitudes and aggressiveness with children (Gollwitzer et al., 2007; Andrews et al., 2010; Lemmer et al., 2015). This research was a necessary first step in determining that IATs modified in form and completed by participants as young as 5 years of age, demonstrate comparable reliability to adult versions of the task.

### Reduced-Length Child-IAT

Researchers often decrease the number of critical trials to create a child-appropriate task (e.g., Rutland et al., 2005; Dunham et al., 2014). Somewhat counter-intuitively given that children have shorter attention spans and are susceptible to fatigue, but consistent with classical test theory (Cronbach, 1951; Nunnally, 1978), the reduced-length Child-IAT (Study 1) did not demonstrate *higher* internal consistency than the first completed traditional-length measure (Study 2). However, both versions demonstrated comparable internal consistencies to what has been found with adults, suggesting that reducing the length of a Child-IAT should not compromise the potential of this measure to provide useful data.

### Repeated Administrations of the Child-IAT

Children may be asked to complete multiple Child-IATs within a single testing session for both theoretical and practical reasons (e.g., Baron and Banaji, 2006; Andrews et al., 2010; Cvencek et al., 2011, 2014). Therefore, we examined the internal consistency of the Child-IAT when completed in succession. Although the first completed Child-IAT demonstrated higher internal consistency than the second measure, all estimates fell within the expected range.

Evidence that responses on the first Child-IAT may be relatively more consistent than responses on subsequently completed tasks can have methodological implications. For example, the results from Study 2 highlight the importance of counterbalancing the order of different Child-IATs to control for erroneous variability that may confound meaningful interpretation of scores. The lower coefficients for the second Child-IAT suggests the need to carefully interpret scores from measures completed in succession. The higher proportion of error variance may attenuate meaningful relationships (Nunnally, 1978; Cunningham et al., 2001) and effect sizes (Baugh, 2002) when multiple Child-IATs are completed in a single testing session. It is also worth noting that comparable reliabilities across both administrations could be due to the fact that children completed the Child-IATs in the presence of a trained experimenter. In cases of response uncertainty, the experimenter would provide a prompt, which could have reduced error variance. Future research will be needed to examine whether Child-IATs are comparably reliable when self-administered by older children or adolescents.

### Test–Retest Reliability

In Study 2 we examined the test–retest reliability of two child-IATs completed within the same testing session using Pearson’s *r* and ICCs. Although less satisfactory than the internal consistency estimates, these coefficients fell within the low end of the range observed in published studies with children and adults. Reconciling acceptable internal consistency with lower test stability is an issue faced by researchers using the IAT. Teige-Mocigemba et al. (2010) have identified two potential explanations: That the IAT (a) captures trait rather than (or in addition to) state associations and/or (b) is sensitive to construct-unrelated response variability (i.e., response strategies, learning effects). The weak test–retest reliability demonstrated by Pearson’s *r* in combination with slightly higher ICCs highlights the possibility that on the Child-IAT children’s and adults’ responses may reflect variability unrelated to their implicit attitudes or be sensitive systematic error (e.g., learning effects, situational cues; Bedard et al., 2000; Weir, 2005) – particularly when completed within the same testing session. Factors contributing to occasion-specific variance, and in particular children’s sensitivity to these factors, is an avenue for future research.

### The Magnitude of Reliability Coefficients

One issue to emerge from this research is the degree to which we should be concerned by the lower internal consistency of implicit as compared to explicit measures. Lower reliability coefficients also emerge with adult responses, indicating that this problem is not restricted to child participants completing Child-IATs. Low reliability of implicit measures is a potential concern as this may reduce the probability of finding group differences (Buchner and Wippich, 2000), which has implications for the effectiveness of the Child-IAT as a tool to assess interventions and relationships between constructs. However, despite lower reliability estimates than what might be expected for explicit measures, race-attitude Child-IATs may be sensitive in capturing differences amongst groups. For example, Asian and White 9- to 12-year-olds exposed to a positive Black exemplar, as compared to positive White exemplar, demonstrated less implicit bias on a Child-IAT (Gonzalez et al., 2016).

Although currently scarce in the literature, we anticipate that evaluating interventions aimed at reducing children’s implicit racial prejudice will be a primary focus of future research. Given that internal consistency may be reduced for subsequently completed child-IATs, we recommend that researchers consider using between-participant designs, as compared to within-participants designs (cf. Schnabel and Asendorpf, 2015), to examine the effectiveness of their interventions. That IAT *D*-scores have been found to meaningfully differentiate between existing and experimental groups for adults (see Teige-Mocigemba et al., 2010, for a review) and children (e.g., Dunham et al., 2007; Gonzalez et al., 2016), perhaps reliability coefficients falling below the generally accepted value of 0.8 should not be interpreted as reflecting a critical deficiency in the Child-IAT (Nunnally, 1978; Pedhazur and Schmelkin, 1991). Implicit measures are subjected to greater sources of error than explicit measures (e.g., momentary inattention, etc.; Buchner and Wippich, 2000; De Houwer, 2003; Fiedler and Bluemke, 2005; Lane et al., 2007), therefore it is perhaps not surprising that responses on the Child-IAT demonstrate greater error variance than what is expected from explicit questionnaires. Indeed, it has been suggested that “low interitem consistency may be a characteristic of response-latency measures more generally” (Cunningham et al., 2001, p. 163), and should perhaps be both expected and accepted. Other methods for calculating reliability and conducting analyses involving the IAT that control for this expected increased measurement error, such as structural equation modeling, may provide more stringent evidence for hypothesis testing (e.g., Cunningham et al., 2001) and should be considered in future research.

It is worth noting that lower internal consistency and test stability are acceptable only to the extent that the Child-IAT is a *valid* measure of the construct of interest. With adults, the race-attitude IAT demonstrates adequate validity (see Lane et al., 2007; Teige-Mocigemba et al., 2010, for reviews), and importantly responses on this measure can be better predictors of behavior during intergroup interactions as compared to self-reported racial bias (Greenwald et al., 2009). Preliminary evidence suggests that responses on Child-IATs may similarly correspond to children’s behavior. Dunham et al. (2011) found that children’s implicit intergroup attitudes predicted the allocation of resources to minimal ingroup members. Although this is a great first step, additional research is needed to address the validity of the race-attitude Child-IAT, with a particular focus on whether this measure can predict children’s spontaneous intergroup behavior (e.g., non-verbal behavior) above and beyond explicitly reported attitudes.

Although the IAT is a widely popular measure that has been used to meaningfully assess children’s social cognition across a range of important topics (see Supplementary Table S1 in Supplementary Materials for a summary), it should be noted that the IAT is not without limitations. For example, the processes underlying the IAT are unclear (see Fiedler et al., 2006; Teige-Mocigemba et al., 2010, for comprehensive reviews) and responses are collapsed into a single relative score of categorical bias, which may mask important differences in the developmental trajectory of implicit attitudes (e.g., Degner and Wentura, 2010; Williams and Steele, unpublished). Therefore, the importance of validating other implicit measures for use with children cannot be understated (Degner and Wentura, 2010; Williams et al., 2016; Williams and Steele, unpublished). We believe that priming tasks and variants of the IAT [i.e., single-category IAT (Karpinski and Steinman, 2006), Go/No Go Association Task (Nosek and Banaji, 2001)] show particular promise in addressing the criticisms of the IAT, and may reveal exciting nuances in the developmental trajectory of children’s implicit social cognition. With this in mind, in the future researchers should aim to select the best measurement tool to assess the impact of prejudice-reduction interventions for improving intergroup relations in childhood.

## Conclusion

Despite decreases in explicitly expressed racial prejudice, people continue to express subtle forms of racial discrimination (Dovidio et al., 2009). A similar pattern is also evident in childhood. Although explicit racial biases decrease around 8-years of age (Raabe and Beelmann, 2011), cross-sectional studies have found implicit racial biases to remain stable across development (see Dunham et al., 2008, for a review). For both adults (Teige-Mocigemba et al., 2010; Nosek et al., 2011) and children (Rutland et al., 2005) decreased explicit racial bias could reflect an unwillingness and/or inability to express such beliefs, instead of an actual decline in underlying race-related associations. Thus to reduce the discrimination and prejudice faced by racial minorities in everyday life, interventions should target implicit attitudes (i.e., Lai et al., 2014) and corresponding behavior (Greenwald et al., 2009; Dunham et al., 2011; Cameron et al., 2012). In order to evaluate the effectiveness of such interventions, researchers require implicit measures with sound psychometric properties. In the current research, we provide evidence that the reliability of the Child-IAT is comparable to the estimates obtained when the traditional IAT is administered to adults. This research provides an important first step, and future research should aim to further assess the validity of this measure, with the goal of determining whether and when implicit racial attitudes can predict consequential outcomes in childhood, including intergroup behavior, peer preferences, and friendships.

## Author Contributions

AW: Data collection, entry, analysis, and interpretation. Study conceptualization and primary role in writing the results for publication. Leading role in addressing reviewers’ comments. JS: Data analysis and interpretation. Study conceptualization and secondary role in writing the results for publication. Secondary role in addressing reviewers’ comments.

## Conflict of Interest Statement

The authors declare that the research was conducted in the absence of any commercial or financial relationships that could be construed as a potential conflict of interest.
