# Supplementary material for: The Reliability of Child-Friendly Race-Attitude Implicit Association Tests
Source: Front Psychol. 2016 Oct 24;7:1576. doi: 10.3389/fpsyg.2016.01576 (PMC5075562; doi:10.3389/fpsyg.2016.01576)
Supplement: Supplementary file 1 [file Data_Sheet_1.docx]

**Supplementary Materials**

**Comprehensive Review of the Internal Consistency of Child-friendly Versions of the IAT**

To provide a comprehensive summary of the internal consistency of child-friendly versions of the traditional Implicit Association Test (Greenwald, McGhee, & Schwartz, 1998) completed exclusively by children, in July 2016 we retrieved articles through PsycINFO searches using the following combination of keywords: *“implicit* AND *child** AND (*attitude** OR *stereotyp**)*”* and *“IAT*  AND *child*”* with restrictions set to peer-reviewed articles with participants less than 18 years, written in English, and published since 1998. Our search returned 57 articles reporting the results of empirical studies that presented results from at least one computer-based child-friendly IAT (Child-IAT) administered to children (< 18 years) using procedures comparable to what is typically used with adults (e.g., Greenwald et al., 1998; Greenwald, Nosek, & Banaji, 2003). Of these articles, just under half (*n* = 23) presented reliability information; this dearth in reporting the internal consistency of Child-IATs further justifies the need for empirical investigations in this area. Please note that although the review of internal consistencies provided in Table S1 may serve as a foundation for our understanding of the psychometric properties of this measure, it is clear that there is still a need for a more systematic examination of the reliability of a race-attitudes Child-IAT.

Table S1

*Internal consistency estimates from published studies with child participants (< 18 years)*

| Authors | Modification | Reliability Method | IAT | Reliability Estimate | Age Group |
| --- | --- | --- | --- | --- | --- |
| Andrews et al. (2010) | Reduced length | Split-half *r* | Smoking vs. healthy food |  |  |
|  |  |  | Time 1 | .54 | 12 years |
|  |  |  | Time 2 | .41 | 12 years |
|  |  |  | Smoking vs. sweets |  |  |
|  |  |  | Time 1 | .37 | 12 years |
|  |  |  | Time 2 | .47 | 12 years |
|  |  |  |  |  |  |
| Bruni & Schultz (2010) | Increased length | Split-half *r* | Environment self-concept |  |  |
|  |  |  | Time 1 | .23 | 10 to 12 years |
|  |  |  | Time 2 | .10 | 10 to 12 years |
|  |  |  |  |  |  |
| Craeynest et al. (2008) |  | α (unspecified) | Food Arousal (Positive) |  |  |
|  |  |  | Study 1 | .56 | 12 to 16 years |
|  |  |  | Study 2 | .55 | 9 to 18 years |
|  |  |  | Food Arousal (Negative) |  |  |
|  |  |  | Study 1 | .55 | 12 to 16 years |
|  |  |  | Study 2 | .54 | 9 to 18 years |
|  |  |  |  |  |  |
| Cvencek et al. (2016) | Reduced length  Picture-based  Auditory words  Response box | α on 20 trial subsets | Gender attitude  Gender identity  Self-esteem | .76  .83  .77 | 5 years  5 years  5 years |

*Table S1 continued*

| Authors | Modification | Reliability Method | IAT | Reliability Estimate | Age Group |
| --- | --- | --- | --- | --- | --- |
| Cvencek, Greenwald, & | Reduced length | α on 2 subscores | Flower/insect attitude | .88 | 4 years |
| Meltzoff (2011) | Picture-based |  | Gender attitude | .85 | 4 years |
|  | Response box |  |  |  |  |
|  |  |  |  |  |  |
| Cvencek et al. (2015) | Reduced length | α on 2 subscores | Math-gender stereotype | > .70 | 7 – 11 years |
|  | Picture-based |  | Math self-concept | > .70 | 7 – 11 years |
|  | Auditory words |  | Gender identity | > .70 | 7 – 11 years |
|  | Response box |  |  |  |  |
|  |  |  |  |  |  |
| Cvencek, Meltzoff, & Greenwald (2011) | Reduced length  Picture-based  Auditory words  Response box | α on 2 subscores | Math-gender stereotype  Math self-concept  Gender identity | .74  .78  .89 | 6 to 10 years  6 to 10 years  6 to 10 years |
| Cvencek et al. (2014) | Reduced length | α on 2 subscores | Math-gender stereotype | .74 | 7 to 11 years |
|  | Picture-based |  | Math self-concept | .80 | 7 to 11 years |
|  | Auditory Words |  | Gender identity | .87 | 7 to 11 years |
|  | Response box |  |  |  |  |
|  |  |  |  |  |  |
| Degner & Wentura | Reduced length | Spearman-Brown *r* | Race attitude | .73 | 9 to 15 years |
| (2010) | Picture-based |  | (German/Turkish) |  |  |
|  |  |  |  |  |  |
| de Jong et al. (2012) |  | Spearman-Brown *r* | Self-esteem | .74 | 13 years |
|  |  |  |  |  |  |
| Dunham et al. (2014) | Reduced length | Split-half *r* | Race attitude | .55 | 6 to 12 years |
|  | Picture-based |  | (Black/Colored) |  |  |
|  |  |  |  |  |  |
| Galdi et al. (2014) | Reduced length | α on 2 subscores | Math-gender stereotype | .84 | 6 years |
|  | Picture-based |  |  |  |  |

*Table S1 continued*

| Authors | Modification | Reliability Method | IAT | Reliability Estimate | Age Group |
| --- | --- | --- | --- | --- | --- |
| Gollwitzer et al. (2007) |  | Guttman | Aggressive self-concept |  |  |
|  |  | split-half | Pretest | .78 | Grades 6 to 8^a^ |
|  |  |  | Posttest | .74 | Grades 6 to 8 |
|  |  |  | Follow up | .74 | Grades 6 to 8 |
|  |  |  |  |  |  |
| Grumm et al. (2011) | Reduced length | Spearman-Brown *r* | Aggressive self-concept | .74 | 9 to 11 years |
|  |  |  |  |  |  |
| Haye et al. (2010) |  | Split-half *r* | Race attitude | .64 | 11 to 15 years |
|  |  |  | (Indigenous/non-Indigenous Chileans) |  |  |
|  |  |  |  |  |  |
| Noel & Thomson | Auditory Words | α on 4 subscores | Alcohol attitude | .83 - .88 | 8 to 14 years |
| (2012) | Response box |  |  |  |  |
|  |  |  |  |  |  |
| Rohner & Bjorklund |  | Spearman-Brown *r* | Homosexuality attitude | .66 | 17 years |
| (2006) |  |  |  |  |  |
|  |  |  |  |  |  |
| Steffens et al. (2010) |  | Split-half *r* | Math-gender stereotype | .80 | 9 years |
|  |  |  |  | .84 | 12, 15 years |
|  |  |  | Math self-concept | .52 | 9 years |
|  |  |  |  | .65 | 12, 15 years |
|  |  |  | Gender identity | .69 | 9 years |
|  |  |  |  | .84 | 12, 15 years |
|  |  |  |  |  |  |
| Suter et al. (2014) |  | Spearman-Brown *r* | Aggression attitude | .89 | 13 to 17 years |
|  |  |  | Transgression attitude | .90 | 13 to 17 years |
|  |  |  | Aggression self-concept | .77 | 13 to 17 years |
|  |  |  | Transgression self-concept | .79 | 13 to 17 years |

*Table S1 continued*

| Authors | Modification | Reliability Method | IAT | Reliability Estimate | Age Group |
| --- | --- | --- | --- | --- | --- |
| Turner et al. (2007) | Picture-based | α on 4 subscores | Race attitude (White/Asian) |  |  |
|  |  |  | Study 1 | .80 | 8 to 11 years |
|  |  |  | Study 2 | .73 | 11 to 15 years |
|  |  |  | Study 3 | .73 | 11 to 15 years |
|  |  |  |  |  |  |
| van Goethem et al. | Reduced length | Spearman-Brown *r* | Bullying attitude |  |  |
| (2010) |  |  | General | .72 | 10 to 13 years |
|  |  |  | Specific | .74 | 10 to 13 years |
|  |  |  |  |  |  |
| Williams et al. (2016) | Reduced length | α on 32 | Flower-insect attitude | .83 | 5 to 10 years |
|  | Picture-based | difference scores | Emotion attitude | .74 | 5 to 10 years |
|  |  |  |  |  |  |
| Zezelj et al. (2015) | Increased length | α (unspecified) | Race attitude |  |  |
|  | Auditory Words |  | (Roma/Other) |  |  |
|  |  |  | Study 1 | .73 | 10 to 11 years |
|  |  |  | Study 2 | .81 | 9 to 11 years |

^a^ Based on the ages provided in Degner & Wentura (2010), it is estimated that participants’ age ranged from 10- to 15-years.

**Internal Consistency Estimates by Age and Race**

As a post-hoc comparison, we examined whether internal consistency would differ by participant age. Consistent with the age groups presented in Study 2 as well as other research examining implicit intergroup attitudes (e.g., Baron & Banaji, 2006; Gonzalez et al., 2016), we first categorized participants as being younger (5- to 7-year-olds) or older (8- to 11-year-olds) and then calculated coefficient alpha on the subblock *D*-scores separately for each age group. The coefficients for both younger (α = .73) and older (α = .71) children were comparable. In an additional exploratory comparison, we examined whether the internal consistency of Black participants, who typically show a different pattern of bias on the Black-White IAT (Newheiser & Olson, 2012), differ from non-Black participants. To accomplish this, we first categorized children as being either Black (identified as Black or Multiracial/Black) or non-Black and calculated coefficient alpha on the subblock *D*-scores separately for each racial group. Regardless of children’s race, we would expect their responses to be consistent across the measure, and this is what we found for both children identified as Black (α = .71) and non-Black (α = .71). Regardless of age and race of the participants, coefficients fell within the range demonstrated in published studies with adults.

**Internal Consistency Estimates by Order of the Critical Blocks**

Previous research has demonstrated block order effects on the IAT, with the target-attribute pairings presented in the first critical blocks interfering with performance on the second critical blocks, especially when the blocks presenting the bias “compatible” trials (i.e., White+positive/Black+negative) are completed first (see Lane et al., 2007; Teige-Mocigemba et al., 2010, for reviews). To examine whether the order in which the critical blocks were completed impacted the internal consistency of the Child-IAT, we extended our analyses presented in the main text to include this as a variable. In both Studies 1 and 2, the order in which the critical blocks were completed did not appear to consistently impact the reliability estimates for this measure, with coefficients ranging from .50 to .89, regardless of the order of the critical blocks. In addition, the test-retest reliability in Study 2 was significant for all order pairings (*r*s > .27, *p* < .05; ICCs > .41), except when the first critical trials were bias “compatible” (White+positive/Black+negative) for the first IAT, and the first critical trials were bias “incompatible” for the second IAT (*r* = .16, *p* = .19; ICC(2,1) = .26). As recommended by Nosek, Greenwald, and Banaji (2007), potential block order effects in *D*-scores should be controlled by counterbalancing the order of critical blocks between participants (see also Nosek et al., 2005), which was done in our studies. Another approach to reduce block order effects involves including extra trials (*n* = 40) in the reverse target discrimination block (Block 5; Nosek et al., 2005). In future administrations of this measure, the potential benefits of adding additional trials in Block 5 for the Child-IAT should be weighed against the potential cost of having children complete a longer task.

**References Reviewed in Table S1**

Andrews, J. A., Hampson, S. E., Greenwald, A. G., Gordon, J., & Widdop, C. (2010). Using the Implicit Association Test to assess children’s implicit attitudes toward smoking. *Journal of Applied Social Psychology, 40,* 2387-2406. doi: 10.1111/j.1559-1816.2010.00663.x

Bruni, C. M., & Schultz, W. (2010). Implicit beliefs about self and nature: Evidence from an IAT game. *Journal of Environmental Psychology, 30,* 95-102. doi: 10.1016/j.jenvp.2009.10.004

Craeynest, M., Crombez, G., Koster, E. H. W., Haerens, L., & De Bourdeaudhuij, I. (2008). Cognitive-motivational determinants of fat food consumption in overweight and obese youngsters: The implicit association between fat food and arousal. *Journal of Behavior Therapy, 39,* 354-368. doi: 10.1016/j.jbtep.2007.09.002

Cvencek, D., Greenwald, A. G., & Meltzoff, A. N. (2016). Implicit measures for preschool children confirm self-esteem’s role in maintaining a balanced identity. *Journal of Experimental Social Psychology, 62,* 50-57. doi: 10.1016/j.jesp.2015.09.015

Cvencek, D., Greenwald, A. G., & Meltzoff, A. N. (2011). Measuring implicit attitudes of 4-year-olds: The preschool Implicit Association Test. *Journal of Experimental Child Psychology, 190,* 187-200. doi: 10.1016/j.jecp.2010.11.002

Cvencek, D., Kapur, M., & Meltzoff, A. N. (2015). Math achievement, stereotypes, and math self-concepts among elementary-school students in Singapore. *Learning and Instruction, 39,* 1-10. doi: 10.1016/j.learninstruc.2015.04.002

Cvencek, D., Meltzoff, A. N., & Greenwald, A. G. (2011). Math-gender stereotypes in elementary school children. *Child Development, 82,* 766-779. doi: 10.1111/j.1467-8624.2010.01529.x

Cvencek, D., Meltzoff, A. N., & Kapur, M. (2014). Cognitive consistency and math-gender stereotypes in Singaporean children. *Journal of Experimental Child Psychology, 117,* 73-91. doi: 10.1016/j.jecp.2013.07.018

Degner, J., & Wentura, D. (2010). Automatic activation of prejudice in children and adolescents. *Journal of Personality and Social Psychology, 98,* 356-374*.* doi: 10.1037/a0017993

de Jong, P. J., Sportel, B. E., de Hullu, E., & Nauta, M. H. (2012). Co-occurrence of social anxiety and depression symptoms in adolescence: Differential links with implicit and explicit self-esteem? *Psychological Medicine, 42,* 475-484. doi: 10.1017/S0033291711001358

Dunham, Y., Newheiser, A., Hoosain, L., Merrill, A., & Olson, K. R. (2014). From a different vantage: Intergroup attitudes among children from low- and intermediate-status racial groups. *Social Cognition, 32*, 1-21. doi: 10.1521/soco.2014.32.1.1

Galdi, S., Cadinu, M., & Tomasetto, C. (2014). The roots of stereotype threat: When automatic associations disrupt girls' math performance. *Child Development, 85,* 250-263. doi: 10.1111/cdev.12128

Gollwitzer, M., Banse, R., Eisenbach, K., & Naumann, A. (2007). Effectiveness of Vienna social competence training on explicit and implicit aggression. *European Journal of Psychological Assessment, 23,* 150-156. doi: 10.1027/1015-5759.23.3.150

Greenwald, A. G., McGhee, D. E., & Schwartz, J. L. K. (1998). Measuring individual differences in implicit cognition: The implicit association test. *Journal of Personality and Social Psychology, 74*, 1464-1480. doi: 10.1037/0022-3514.74.6.1464

Greenwald, A. G., Nosek, B. A., & Banaji, M. R. (2003). Understanding and using the implicit association test: I. An improved scoring algorithm. *Journal of Personality and Social Psychology, 85*, 197-216. doi: 10.1037/0022-3514.85.2.197

Grumm, M., Hein, S., & Finderle, M. (2011). Predicting aggressive behavior in children with help of measures of implicit and explicit aggression. *International Journal of Behavioral Development, 35,* 352-357. doi: 10.1177/0165025411405955

Haye, A., Gonzalez, R., Ordonez, G., Bohner, G., Siebler, F., Sirlopu, D., … Torres, D. (2010). System-perpetuating asymmetries between explicit and implicit intergroup attitudes among indigenous and non-indigenous Chileans. *Asian Journal of Social Psychology, 13,* 163-172. doi: 10.1111/j.1467-839X.2010.01311.x

Noel, J. G., & Thomson, N. R. (2012). Children's alcohol cognitions prior to drinking onset: Discrepant patterns from implicit and explicit measures. *Psychology of Addictive Behaviors, 26,* 451-459. doi: 10.1037/a0025531

Rohner, J. C., & Bjorklund, F. (2006). Do self-presentation concerns moderate the relationship between implicit and explicit homonegativity measures? *Scandinavian Journal of Psychology, 47,* 379-385. doi: 10.1111/j.1467-9450.2006.00522.x

Steffens, M. C., Jelenec, P., & Noack, P. (2010). On the leaky math pipeline: Comparing implicit math-gender stereotypes and math withdrawal in female and male children and adolescents. *Journal of Educational Psychology, 102,* 947-963. doi: 10.1037/a0019920

Suter, M., Pihet, S., de Ridder, J., Zimmermann, G., & Stephan, P. (2014). Implicit attitudes and self-concepts towards transgression and aggression: Differences between male community and offender adolescents, and associations with psychopathic traits. *Journal of Adolescence, 37,* 669-680. doi: 10.1016/j.adolescence.2014.03.004

Turner, R. N., Hewstone, M., & Voci, A. (2007). Reducing explicit and implicit outgroup prejudice via direct and extended contact: The mediating role of self-disclosure and intergroup anxiety. *Journal of Personality and Social Psychology, 93*, 369-388. doi: 10.1037/0022-3514.93.3.369

van Goethem, A. A. J., Scholte, R. H. J., & Wiers, R. W. (2010). Explicit- and implicit bullying attitudes in relation to bullying behavior. *Journal of Abnormal Child Psychology, 38,* 829-842. doi: 10.1007/s10802-010-9405-2

Williams, A., Steele, J. R., & Lipman, C. (2016). Assessing children’s implicit attitudes using the Affect Misattribution Procedure. *Journal of Cognition and Development, 17,* 505-525. doi: 10.1080/15248372.2015.1061527

Zezelj, I., Jaksic, I., & Josic, S. (2015). How contact shapes implicit and explicit preferences: Attitudes toward Roma children in inclusice and non-inclusive environment. *Journal of Applied Social Psychology, 45,* 263-272. doi: 10.1111/jasp/12293
